# The Influence of Factors such as Anxiety on the White Coat Effect during the Treatment of Patients with Hypertension

**DOI:** 10.31083/j.rcm2311359

**Published:** 2022-10-25

**Authors:** Dengyue Xu, Hengxia Qiu, Ze Li, Peishi Yan, He Xu, Yu Gu, Hailong Lin

**Affiliations:** ^1^Postgraduate College, China Medical University, 110122 Shenyang, Liaoning, China; ^2^Department of Cardiology, Dalian Municiple Central Hospital, 116000 Dalian, Liaoning, China; ^3^Department of Cadre Ward, 79th Group Army Hospital of PLA Army, 111000 Liaoyang, Liaoning, China; ^4^Geriatrics Center, Dalian Municiple Friendship Hospital, 116000 Dalian, Liaoning, China

**Keywords:** hypertension, white coat effect, Self-Rating Anxiety Scale, Beck Anxiety Inventory, blood pressure

## Abstract

**Background:**

The white coat effect is observed in many patients with hypertension, but its mechanism is still unclear and anxiety is often thought to be a key point.

**Methods:**

A total of 544 patients who met the inclusion criteria were recruited through outpatient clinics. Three months after systematic treatment, the office blood pressure and ambulatory blood pressure monitoring (ABPM) were examined. Patients who reached the ABPM standard were divided into white coat effect (n = 112) and control (n = 432) groups according to the results of the office blood pressure. The degree of anxiety in the two groups was evaluated using the Self-rating Anxiety Scale (SAS) and the Beck Anxiety Scale (BAI). Differences in anxiety, gender, age, number of antihypertensive drugs, cost per tablet and marital status were analyzed.

**Results:**

There was no significant difference in the degree of anxiety between the white coat and control groups, with mean SAS standard scores of 32.8 ± 8.5 *vs*. 31.8 ± 9.9, respectively (*p* = 0.170). Similarly, the mean BAI standard scores were 31.4 ± 8.3 *vs*. 31.2 ± 9.5, respectively (*p* = 0.119). Logistic regression analysis showed that the factors of female gender (β = –1.230, *p *< 0.001), old age (β = 0.216, *p *< 0.001), number of antihypertensive drugs (β = 1.957, *p *< 0.001), and cost per tablet (β = 1.340, *p *< 0.001) were significantly related to the white coat effect.

**Conclusions:**

Anxiety was not necessary for the white coat effect in hypertension patients during treatment. Female gender, old age, number of antihypertensive drugs used and cost per tablet were related to the white coat effect in hypertension patients during treatment.

## 1. Introduction

The clinical diagnosis of hypertension is usually based on office blood pressure (OBP). However, it is well-known that blood pressure (BP) in the clinic is related not only to the basic BP of the individual, but that it may also be influenced by neuropsychological changes during the patient’s treatment process [1]. The condition whereby the BP measured at home or the ambulatory BP is lower than the OBP is referred to as white coat hypertension. The difference between OBP and out-of-office BP in white-coat hypertension is referred to as the white coat effect. It is considered clinically significant when the OBP is >20/10 mmHg higher than the out-of-office BP [2]. The incidence of the white coat effect can be as high as 30% in hypertensive patients [3, 4], although the mechanism remains unclear. There has been much work to suggest that psychological factors may be related to the occurrence of the white coat effect [5, 6, 7], but no definite conclusions have so far been drawn. In the present study, patients with essential hypertension stratified by white coat effect and who had received antihypertensive therapy were selected as subjects for investigation. The relationship between factors, such as anxiety, and white coat effect in essential hypertension patients during treatment was investigated through detailed questionnaires.

## 2. Patients and Methods

### 2.1 Inclusion and Exclusion Criteria

Approval for this study was obtained from the Ethics Committee of the Dalian Municipal Central Hospital (YN2017-042-01). All patients with essential hypertension from September 2017 to September 2020 were enrolled in the Cardiology Outpatient department, Dalian Central Hospital, affiliated to Dalian Medical University. Inclusion criteria were: (1) Age range from 18 to 80 years; (2) Newly diagnosed with essential hypertension. The diagnostic criteria for essential hypertension were in accordance with 2010 Chinese guidelines for the management of hypertension [8]. The office BP before treatment was ≥140/90 mmHg (1 mmHg = 0.133 kpa), and the home blood pressure monitoring (HBPM) was ≥135/85 mmHg, or the 24 h mean BP of ambulatory blood pressure monitoring (ABPM) was ≥130/80 mmHg, with a daytime mean BP of ABPM ≥135/85 mmHg, and a night mean BP of ABPM of ≥120/70 mmHg. (3) The patient agreed to participate in the study and signed informed consent. Exclusion criteria [9]: (1) Individuals with obvious intellectual, hearing or physical impairment who was unable to cooperate. (2) Patients with a family history of mental illness. (3) Patients with malignant tumors. (4) Patients with cardiac insufficiency. (5) Individuals with recent use of psychoactive drugs. (6) Individuals who recently suffered a family tragedy. (7) Patients with occult hypertension, refractory hypertension, stroke, coronary heart disease, severe anemia, severe liver, and secondary hypertension, including obstructive sleep apnoea, renal parenchymal disease, atherosclerotic renovascular disease, fibromuscular dysplasia, primary aldosteronism, phaeochromocytoma, Cushing’s syndrome, thyroid disease, hyperparathyroidism and coarctation of the aortic. (8) Patients with severe trauma and a surgical history in the previous six months.

### 2.2 Treatment Process Before Grouping

All patients were given regular and systematic drug therapy for 3 months in accordance with the requirements of the 2010 Chinese guidelines [8] the 2013 European Society of Hypertension (ESH)/European Society of Cardiology (ESC) guidelines [10] and 2018 ESC/ESH guidelines [11] for the management of arterial hypertension. The addition or subtraction of medications had been stopped for at least a month. Follow-up visits were made in the outpatient department every two weeks. The increase or decrease in drug treatment was based on the OBP, HBPM and patient symptoms. The increase or adjustment of drugs was stopped whenever BP in the clinic was <140/90 mmHg or HBPM was <135/85 mmHg, or when excessive hypotensive symptoms such as dizziness and fatigue appeared. The combination drugs were in single-drug dosage forms, and the dosage used was the internationally accepted conventional dosage. Non-pharmacological treatments, such as lifestyle changes, were also recommended for patients.

### 2.3 Methods of Determining OBP

The method recommended by the 2010 Chinese guidelines for the management of hypertension [8] was used to measure the sitting BP of subjects in the morning. This was performed with a standard desktop mercury sphygmomanometer (30704005, Yuwell Group, Jiangsu, China) regularly calibrated by a professional doctor in a consulting room.

### 2.4 ABPM Methods

A Spacelabs Healthcare Automatic 24 h Ambulatory Blood Pressure Monitor (90217, Spacelabs Healthcare Ltd. Seattle, Washington, USA) was used, with standardized measurement performed according to the 2010 Chinese guidelines for the management of hypertension [8, 12]. If the invalid pseudo-error and blank data were found to exceed 30% of the total number, the data was considered invalid and measurements were made again the next day. The patient’s daily life and treatment process remained unchanged during monitoring, with the left upper limb remaining relatively stationary during each measurement.

### 2.5 HBPM Methods

The upper arm electronic sphygmomanometer (HEM7136, OMRON Corporation, Kyoto, Japan) was used in accordance with the 2010 Chinese guidelines for the management of hypertension [8]. Patients were asked to measure BP every morning and evening, after sitting for 5 to 10 minutes before measurement. BP was measured in the sitting position in order to keep the sphygmomanometer and heart at the same level during measurement. The measurement was performed 2 or 3 times repeatedly and the average value was taken. The BP from each measurement was recorded in detail.

### 2.6 Grouping

Subjects were divided into two groups according to the OBP after 3 months of treatment: (1) The essential hypertension group with the white coat effect (white coat effect group). In these subjects, the difference between the systolic blood pressure (SBP) in the office and the 24 h mean SBP was 20 mmHg or more, and the difference between the diastolic blood pressure (DBP) in the office and the 24 h mean DBP was 10 mmHg or more. (2) The essential hypertension group without the white coat effect (control group). In these subjects, the difference between the SBP in the office and the 24 h mean SBP was less than 20 mmHg, and the difference between the DBP in the office and the 24 h mean DBP was less than 10 mmHg.

### 2.7 Data Collection

The data including gender, age, ethnicity, marital status, number of antihypertensive drugs used, and average daily cost of antihypertensive drugs was collected by uniformly trained professionals in accordance with uniform collection procedures through the uniform epidemiological questionnaires.

### 2.8 Anxiety Scale Questionnaire

The Self-rating Anxiety Scale (SAS) and Beck Anxiety Inventory (BAI) were used to determine the level of anxiety. The survey was conducted in the form of a questionnaire by the same trained medical staff and was given in a quiet special room. The purely academic purpose of the survey was fully explained to the patients before the survey, so as to eliminate the patients’ defensive psychology. After seeking the consent of patients, the two scales were filled in by the patients themselves, and a few patients with a low educational level and/or poor vision were assessed independently according to their own and assisted answers. Before taking the questionnaire, how to fill in the scale correctly and the meaning of each item were explained to each patient, and then they were asked to fill in the questionnaire by making an independent self-assessment of their actual feelings of the last week. Furthermore, all patients had been informed of their OBP and ambulatory BP results before the investigation.

### 2.9 Assessment Criteria of Anxiety State 

The SAS [13, 14] contains 20 items that measure the frequency of anxiety symptoms. The score was divided into four levels: 1 (no or little time), 2 (a small part of the time), 3 (a considerable amount of time), and 4 (most or all of time), and corresponding to 1, 2, 3, or 4 points, respectively. The score of items 5, 9, 13, 17, and 19 were in reverse, and the other items were scored in sequence, as shown in Appendix Table 5. The accumulated score of each item was the raw score. After the following formula conversion, Y = int (1.25x), in which the raw score was multiplied by 1.25 and the integral part was taken, the standard score was calculated. The anxiety severity was defined as follows: a score of 25–49 represented no anxiety; a score of 50–59 represented mild anxiety; a score of 60–69 represented moderate anxiety; and a score of 70–100 represented severe anxiety. The national normal mean of raw scores was 29.78 ± 0.46 (n = 1158), and the upper limit of the total raw score was 40.

The BAI [14] includes 21 different anxiety symptoms and mainly evaluates the extent to which subjects are bothered by different anxiety symptoms. The subject filling in the questionnaire was required to select the extent to which each symptom bothered them, which was divided into 1 point, representing no influence, 2 points, representing mild influence, 3 points, representing moderate influence, and 4 points, representing serious influence (Appendix Table 6). The scores of all items were accumulated to obtain raw scores, which were then converted into standard scores using the formula: Y = int (1.19x) and taking the integral part. The standard score of 45 was the threshold of judgment, and the higher the score, the more anxious the individual.

Both scales were used, and anxiety was confirmed when the scores were higher than their standard scores.

### 2.10 Statistical Analysis

SPSS Statistics (version 24.0, SPSS Statistics, International Business Machines Corporation, Armonk, NY, USA) was used for statistical analysis. The measurement data were presented as the mean ± standard deviation (SD). If they were normally distributed, a *t* test was used for comparison between groups. Otherwise, a nonparametric rank sum test was used. The counting data were presented as rates or composition ratios, and a Chi-square test or Fisher’s exact probability method was used for comparison between groups. A logistic regression model was used to analyze other possible influencing factors of the white coat effect. If the *p* value was <0.05, it was considered statistically significant.

## 3. Results

**Fig. 1.**
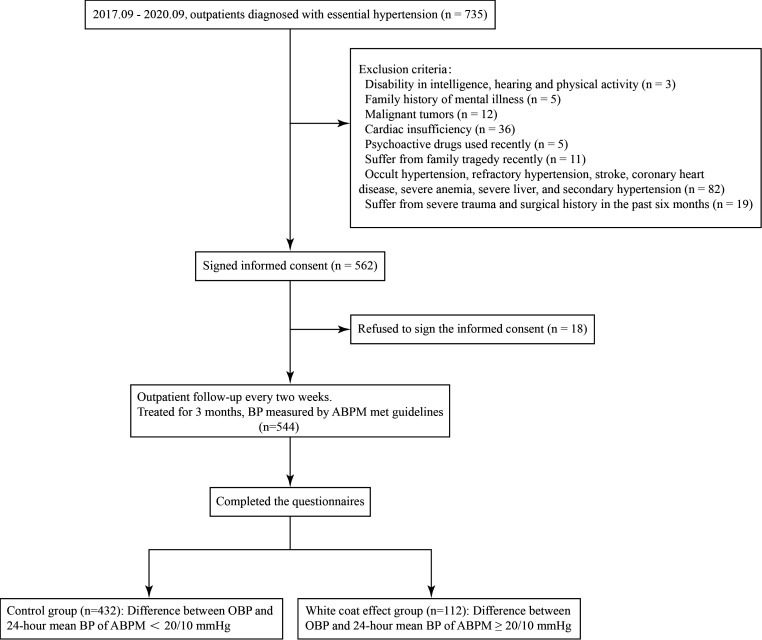
**Flowchart of the study**. BP, blood pressure; ABPM, ambulatory blood pressure monitoring; OBP, office blood pressure.

As shown in Fig. 1, 432 patients (79.4%) in the control group and 112 patients (20.6%) in the white coat effect group were enrolled. All of them completed the questionnaire survey, with the recovery rate of 100%.

### 3.1 Comparison of BP Changes 

There was no significant difference in office SBP (169.1 ± 5.5 mmHg* vs*. 168.6 ± 6.2 mmHg, *p* = 0.433) and DBP (111.3 ± 5.5 mmHg *vs*. 111.0 ± 4.5 mmHg,* p* = 0.574) between the two groups before initiation of treatment. After treatment, the office BP in the white coat effect group was significantly higher than that in the control group. The difference of SBP between them was (29.7 ± 5.3) mmHg, and that of DBP was (14.4 ± 4.2) mmHg. The office BP of the white coat group was significantly higher than the value of ABPM after treatment. The difference between office SBP and 24 h mean SBP in the white coat effect group was (42.9 ± 8.0) mmHg, and that in the control group was (15.2 ± 3.1) mmHg. The difference between them was statistically significant (*p <* 0.001). The difference between office DBP and 24 h mean DBP in the white coat effect group was (21.1 ± 6.8) mmHg, and that in the control group was (7.3 ± 1.9) mmHg. Likewise, the difference between them was statistically significant (*p <* 0.001) (for details see Appendix Table 7).

### 3.2 Comparison of Anxiety Degree 

The highest raw score of SAS in the control group was 58, and the lowest score was 20, with an average of 25.7 ± 8.0. The highest standard score was 72, the lowest was score 25, and the average score was 31.8 ± 9.9. Among them, there were 48 cases with a standard score of ≥50. The highest raw score of SAS in the white coat effect group was 50, and the lowest score was 20, with an average score of 26.2 ± 6.8. The highest standard score was 63, the lowest score was 25, and the average score was 32.8 ± 8.5. Among them, there were 11 cases that had a standard score of ≥50. There was no statistically significant difference between the white coat effect group and the control group in either raw score or standard score (Table 1).

**Table 1.** **The score of SAS and BAI ($\bar{x}$ ± s )**.

Groups	SAS Score				BAI Score			
	Raw	*p*	Standard	*p*	Raw	*p*	Standard	*p*
Control group (n = 432)	25.7 ± 8.0	0.137	31.8 ± 9.9	0.170	26.7 ± 7.9	0.126	31.2 ± 9.5	0.119
White coat effect group (n = 112)	26.2 ± 6.8		32.8 ± 8.5		26.9 ± 7.0		31.4 ± 8.3	

SAS, Self-Rating Anxiety Scale; BAI, Beck Anxiety Inventory.

The highest raw score of BAI in the control group was 58, and the lowest score was 21, with an average of 26.7 ± 7.9. The highest standard score was 69, the lowest was score 24, and the average score was 31.2 ± 9.5. Among them, there were 48 cases that had a standard score ≥45, and all of them had an SAS standard score ≥50. The highest raw score of BAI in the white coat effect group was 51, and the lowest score was 21, with an average of 26.9 ± 7.0. The highest standard score was 60, the lowest score was 24, and the average score was 31.4 ± 8.3. Among them, there were 11 cases that had a standard score ≥45, and all of them had an SAS standard score ≥50. There was no statistically significant difference between the white coat effect group and the control group (*p *> 0.05) for either the raw score or standard score (Table 1).

### 3.3 Comparison of Other Clinical Factors

The respondents were all Han Chinese, and the medication use was summarized in the Appendix Table 8. The proportion of females, age, daily cost of antihypertensive drugs, and number of antihypertensive drugs in the white coat effect group were significantly higher than those in the control group (*p <* 0.05). However, there was no statistically significant difference in the marital status between the two groups (*p >* 0.05) (Table 2).

**Table 2.** **Characteristics of the study population ($\bar{x}$ ± s )**.

Items		Control group	White coat effect group	*p*
		(n = 432)	(n = 112)	
Gender				
	Male (n, %)	243 (56.3)	24 (21.4)	<0.001
	Female (n, %)	189 (43.7)	88 (78.6)	
Age (years)		56.6 ± 7.4	67.4 ± 5.3	<0.001
Number of antihypertensive drugs (n, %)				
	1	21 (4.9)	0	<0.001
	2	104 (24.1)	0	
	3	214 (49.5)	42 (37.5)	
	4	93 (21.5)	70 (62.5)	
Daily cost of antihypertensive drug (yuan)		4.6 ± 1.9	7.7 ± 2.4	<0.001
Cost per tablet (yuan)		1.6 ± 0.7	2.2 ± 0.7	<0.001
Marital status (n, %)				
	Unmarried	0	1 (0.9)	0.058
	Married	417 (96.5)	104 (92.8)	
	Widowed	15 (3.5)	7 (6.3)	

### 3.4 Logistic Regression Analysis

With the white coat effect as the dependent variable, and anxiety, gender, age, daily cost of antihypertensive drugs, number of antihypertensive drugs, and marital status as independent variables, logistic regression analysis was performed (Table 3). The results showed that gender, age, number of antihypertensive drugs and cost per tablet were factors related to the white coat effect (Table 4).

**Table 3.** **Quantification and assignment for risk factors of white coat effect**.

Risk Factors	Variables	Assignment
Anxiety	$X_{1}$	“0” for non-anxiety, “1” for anxiety
Gender	$X_{2}$	“0” for female, “1” for male
Age	$X_{3}$	Specific values
Number of antihypertensive drugs	$X_{4}$	Specific values
Cost per tablet	$X_{5}$	Specific values
Marital status	$X_{6}$	“0” for unmarried, “1” for married, “2” for widowed
White coat effect	Y	“0” for non-white coat effect, “1” for white coat effect

**Table 4.** **Logistic regression analysis of risk factors of white coat effect (n = 544)**.

Risk Factors	B	SE	Wald	*p*	OR	95% CI
Anxiety	0.313	0.554	0.318	0.573	1.367	0.461–4.049
Gender	–1.230	0.347	12.579	<0.001	0.292	0.148–0.577
Age	0.216	0.027	66.136	<0.001	1.241	1.178–1.307
Number of antihypertensive drugs	1.957	0.282	47.974	<0.001	7.075	4.067–12.307
Cost per tablet	1.340	0.228	34.601	<0.001	3.820	2.444–5.971
Marital status	–1.139	0.656	3.015	0.082	0.320	0.089–1.158

B, regression coefficient; SE, standard error; OR, odds ratio; CI, confidence interval.

## 4. Discussion

In the present study, we found that there was no significant difference in anxiety status between the white coat effect group and the control group. However, gender, age, number of antihypertensive drugs used and coat per tablet were the influence factors of white coat effect in hypertensive patients during treatment.

The concept of the white coat effect was described precisely by Mancia *et al*. in 1983 [15]. When a patient was undergoing traumatic intra-arterial ambulatory BP monitoring in the consulting room, a doctor in a white coat entered the room, then the patient’s BP suddenly rose rapidly and was at its highest point within 4 minutes, with an average increase of 27/14 mmHg. The BP gradually dropped within 10 minutes. The term white coat hypertension was originally limited to untreated individuals but has been extended to patients under antihypertensive treatment in whom only OBP has not been treated to target, with the term white coat uncontrolled hypertension (WUCH), compared with sustained uncontrolled hypertension (SUCH) [11]. The white coat effect is used to describe the difference between an elevated OBP and a lower home or ambulatory BP in both untreated and treated patients.

The specific mechanism of the white coat effect is not clear, and it is speculated to be related to a stress response. According to Kai *et al*. [16] and Pioli *et al*. [17], when patients received pressure measurements by medical staff in the consulting room, the scenario was likely to cause a stress and alert response in patients, resulting in excessive tension and activation of the sympathetic catecholamine system, leading to an increase in BP in the clinic. Perhaps the words of the medical staff might also affect the measured BP [18]. Smith *et al*. [19] and Caini *et al*. [20] did find that activity of the sympathetic nervous system and renin-angiotensin system were increased in patients with the white coat effect. There are obvious individual differences in stress responses, which are related to individual neurologic characteristics. Anxiety is a common neurological personality trait. Previous studies [21, 22, 23] have shown that anxiety patients are prone to hypertension.

Peiliang *et al*. [24] and Cobos *et al*. [25] have found that anxiety might be related to the white coat effect, but this study did not support this possibility. This study did not find that hypertensive patients with the white coat effect were more anxious than hypertensive patients without the white coat effect. Moreover, it was also found that the anxiety score of all hypertensive patients included in the study was not higher than the constant index of the normal population, which was different from previous studies [26, 27]. First, in previous studies, anxiety assessment was mostly carried out before treatment, while the anxiety assessment in this study was conducted after 3 months of antihypertensive treatment. In addition to specialized drug treatments for hypertensive patients, non-drug treatment including general psychological counseling was emphasized in the study. Although the psychological counseling was not professional, it was speculated that it might play a role. Second, the authoritative personnel in the hypertension outpatient clinic might provide a strong psychological comfort to patients. The professional capacities of medical staff provided patients confidence in the diagnosis and treatment of hypertension during the follow-up visit. This study showed that anxiety was not necessary for the white coat effect. Hypertensive patients without anxiety could also have a transient, hidden mood fluctuation after entering the clinic, which triggers a neuroendocrine reaction and temporarily increases BP, which is the white coat effect.

Furthermore, this study also analyzed the relationship between gender, age, number of antihypertensive drugs, cost per tablet, and marital status, and the white coat effect of treated hypertensive patients. This study found that BP before treatment and marital status had nothing to do with the white coat effect, but females and elderly patients were more likely to have the white coat effect, which was consistent with other research reports [28, 29]. Moreover, this study also found that both the number of antihypertensive drugs and the cost per tablet were related to the white coat effect, namely, the more antihypertensive drugs used, the higher cost per tablet spent, the greater the possibility that the white coat effect occurred. The mechanism of the white coat effect in women is not clear; it may be related to female hormones. Oyola *et al*. [30] found that women were more prone to exaggerated response to stress, due to the enhanced activity of hypothalamo-pituitary-adrenal (HPA) axis resulting from the increased level of estradiol under stress. However, this proposition has been disputable, and needs to be further studied [31]. It has long been reported [32, 33] that untreated elderly patients were prone to the white coat effect, and this study showed that the white coat effect in hypertensive patients with effective antihypertensive treatment was also related to age. The reasons why the white coat effect was likely to occur in the elderly might be as follows. On the one hand, the mental sensitivity of elderly patients to diseases was generally enhanced [34], which easily triggers neuroendocrine reactions. On the other hand, the elasticity of the aorta in elderly patients is decreased, which reduced the buffering ability to the increasing pressure because of the change in cardiac stroke volume. When psychological stress led to an increase in cardiac stroke volume, the increase in SBP was more pronounced. The relationship between the number of antihypertensive drugs and the cost per tablet and the white coat effect might be related to the following aspects: First, the increase or decrease of drugs based on OBP might lead to overuse of antihypertensive drugs in hypertensive patients with the white coat effect. The home BP was referred to when adding or reducing drugs, and the 24 h ambulatory BP level of the white coat effect group was not lower than that of the control group, but this possibility cannot be completely ruled out. Second, it meant that BP was difficult to control, and the condition was serious when the number of antihypertensive drugs used was large and the cost per tablet was high, which could cause greater psychological impact on the pressure measurement of patients in the office. Third, patients who used multiple antihypertensive drugs with a high cost and with difficulty in controlling BP might have a stronger rapid pressor reflex mechanism, so that the white coat effect was more obvious.

## 5. Limitations

This study was a single-center study, which has its inherent limitations. The small sample size may have led to sampling deviations. Because of geographical limitations, the patients in this study were Han Chinese, and the white coat effect of other ethnicities was not analyzed. The white coat effect of patients before antihypertensive treatment was not analyzed, which may cause aberrations in the analysis of the results after treatment. In short, more comprehensive research is needed in the future.

## 6. Conclusions

Anxiety may not be the cause of the white coat effect in patients with hypertension during treatment. Female, old age, number of antihypertensive drugs used, and cost per tablet were related to the white coat effect in hypertension patients during treatment. Clinically, attention should be paid to the identification of the white coat effect in the diagnosis and treatment of hypertension, and the treatment plan should be adjusted according to the situation.

## Appendix

See Tables 5,6,7,8.

**Table 5.** **Details of Self-Rating Anxiety Scale (SAS)**.

Items	No or very little time	A small part of time	A considerable amount of time	Most or all of the time
1. I feel more nervous or anxious than usual.	1	2	3	4
2. I’m afraid for no reason.	1	2	3	4
3. I easily get upset or frightened.	1	2	3	4
4. I think I might go crazy.	1	2	3	4
*5. I think everything is fine.	4	3	2	1
6. My hands and feet trembled.	1	2	3	4
7. I suffer from headaches, neck pains and backaches.	1	2	3	4
8. I feel weak and tired easily.	1	2	3	4
*9. I feel calm and I tend to sit still.	4	3	2	1
10. I feel my heart beating fast.	1	2	3	4
11. I am troubled by bouts of dizziness.	1	2	3	4
12. I have seizures or feelings of fainting.	1	2	3	4
*13. I breathe in and out easily.	4	3	2	1
14. I have numbness and tingling in my hands and feet.	1	2	3	4
15. I am troubled with stomachache and indigestion.	1	2	3	4
16. I often have to urinate.	1	2	3	4
*17. My hands and feet are often dry and warm.	4	3	2	1
18. I’m flushed and hot.	1	2	3	4
*19. I fall asleep easily and get a good night’s sleep.	4	3	2	1
20. I have nightmares.	1	2	3	4

*The score of items 5, 9, 13, 17 and 19 are in reverse.

**Table 6.** **Details of Beck Anxiety Inventory (BAI)**.

Items	None	Mild	Moderate	Severe
		Not much disturbance	Feel uncomfortable but tolerable	Can barely endure
1. Numbness or tingling	1	2	3	4
2. Feel hot	1	2	3	4
3. The legs tremble	1	2	3	4
4. Can’t relax	1	2	3	4
5. Fear of something bad happening	1	2	3	4
6. Dizziniess	1	2	3	4
7. Palpitations or increased heart rate	1	2	3	4
8. Distracted	1	2	3	4
9. Frightened	1	2	3	4
10. Nervous	1	2	3	4
11. Choked	1	2	3	4
12. The hands tremble	1	2	3	4
13. Shake	1	2	3	4
14. Afraid of out of control	1	2	3	4
15. Dyspnea	1	2	3	4
16. Fear of dying	1	2	3	4
17. Panic	1	2	3	4
18. Indigestion or abdominal discomfort	1	2	3	4
19. Fainting	1	2	3	4
20. Face redness	1	2	3	4
21. Sweating (not due to heat)	1	2	3	4

**Table 7.** **Changes of BP ($\bar{x}$ ± s ) (mmHg)**.

BP		Control group	White coat effect group	*p*
		(n = 432)	(n = 112)	
OBP before treatment				
	SBP	168.6 ± 6.2	169.1 ± 5.5	0.433
	DBP	111.0 ± 4.5	111.3 ± 5.5	0.574
OBP after treatment				
	SBP	131.2 ± 3.2	160.9 ± 6.1	<0.001
	DBP	81.7 ± 2.3	96.2 ± 4.7	<0.001
24 h mean BP after treatment				
	SBP	116.0 ± 3.8	118.0 ± ${\mathrm{5.4}}^{a}$	<0.001
	DBP	74.5 ± 2.7	75.1 ± ${\mathrm{5.2}}^{a}$	0.231
Daytime mean BP after treatment				
	SBP	120.7 ± 4.1	122.5 ± ${\mathrm{4.9}}^{a}$	<0.001
	DBP	78.1 ± 3.1	77.2 ± ${\mathrm{4.3}}^{a}$	0.031
The difference between OBP and 24 h mean BP after treatment				
	SBP	15.2 ± 3.1	42.9 ± 8.0	<0.001
	DBP	7.3 ± 1.9	21.1 ± 6.8	<0.001
The difference between OBP and daytime mean BP after treatment				
	SBP	10.5 ± 4.2	38.4 ± 7.1	<0.001
	DBP	3.6 ± 2.7	19.0 ± 6.0	<0.001

24 h mean BP and daytime mean BP after treatment of the white coat effect group, compared with the office BP after treatment of the same group, ^a^*p *< 0.05. BP, blood pressure; OBP, office blood pressure; SBP, systolic blood pressure; DBP, diastolic blood pressure.

**Table 8.** **Number of patients taking each medication**.

Medication	Control group	White coat effect group	*p*
	(n = 432)	(n = 112)	
Nifedipine (Bayer, 30 mg)	30 (6.9%)	35 (31.3%)	<0.001
Amlodipine (Pfizer, 5 mg)	21 (4.9%)	28 (25.0%)	<0.001
Amlodipine (Landi, 5 mg)	177 (41.0%)	21 (18.8%)	<0.001
Lacidipine (Sanchine, 4 mg)	126 (29.2%)	27 (24.1%)	0.289
Benazepril (Novartis, 10 mg)	80 (18.5%)	13 (11.6%)	0.083
Perindopril (Acertil, 4 mg)	45 (10.4%)	13 (11.6%)	0.716
Irbesartan (Coaprovel, 150 mg)	64 (14.8%)	18 (16.1%)	0.740
Telmisartan (Boehringer Ingelheim, 80 mg)	8 (1.9%)	25 (22.3%)	<0.001
Telmisartan (CCPC, 80 mg)	87 (20.1%)	9 (8.0%)	0.003
Bisoprolol (Merck, 5 mg)	71 (16.4%)	20 (17.9%)	0.719
Metoprolol (AstraZeneca, 25 mg)	239 (55.3%)	66 (58.9%)	0.493
Hydrochlorothiazide (Yunpeng, 25 mg)	94 (21.8%)	64 (57.1%)	<0.001
Irbesartan Hydrochlorothiazide* (Coaprovel, 150 mg/12.5 mg)	69 (16.0%)	15 (13.4%)	0.501
Telmisartan Hydrochlorothiazide* (CCPC, 40 mg/12.5 mg)	31 (7.2%)	19 (17.0%)	0.001

*When calculating the number of drugs, one compound preparation was counted as two drugs. CCPC, Suzhou Chung-Hwa Chemical & Pharmaceutical industrial CO., LTD.

## References

[1] Smolensky MH, Hermida RC, Portaluppi F (2017). Circadian mechanisms of 24-hour blood pressure regulation and patterning. *Sleep Medicine Reviews*.

[2] Whelton PK, Carey RM, Aronow WS, Casey DE, Collins KJ, Dennison Himmelfarb C (2018). 2017 ACC/AHA/AAPA/ABC/ACPM/AGS/APhA/ASH/ASPC/NMA/PCNA Guideline for the Prevention, Detection, Evaluation, and Management of High Blood Pressure in Adults: A Report of the American College of Cardiology/American Heart Association Task Force on Clinical Practice Guidelines. *Hypertension*.

[3] Siddiqui M, Judd EK, Oparil S, Calhoun DA (2017). White-Coat Effect is Uncommon in Patients with Refractory Hypertension. *Hypertension*.

[4] Dingli X, Yuli H (2020). Clinical detection and management of white coat hypertension. *Chinese Journal of Hypertension*.

[5] Nuredini G, Saunders A, Rajkumar C, Okorie M (2020). Current status of white coat hypertension: where are we. *Therapeutic Advances in Cardiovascular Disease*.

[6] Ogedegbe G (2010). Causal mechanisms of masked hypertension: socio-psychological aspects. *Blood Pressure Monitoring*.

[7] Terracciano A, Scuteri A, Strait J, Sutin AR, Meirelles O, Marongiu M (2014). Are personality traits associated with white-coat and masked hypertension. *Journal of Hypertension*.

[8] Lisheng L (2011). 2010 Chinese guidelines for the management of hypertension. *Chinese Journal of Hypertension*.

[9] Hui C, Jun W, Xiaoping X, Hongzhen X, Yanjun W, Shasha L (2016). Effect of flupentixol and melitracen on white coat effect and blood pressure circadian rhythm in white coat hypertensive patients. *Chinese Journal of Cardiovascular Medicine*.

[10] Mancia G, Fagard R, Narkiewicz K, Redón J, Zanchetti A, Böhm M (2013). 2013 ESH/ESC Guidelines for the management of arterial hypertension: the Task Force for the management of arterial hypertension of the European Society of Hypertension (ESH) and of the European Society of Cardiology (ESC). *Journal of Hypertension*.

[11] Williams B, Mancia G, Spiering W, Agabiti Rosei E, Azizi M, Burnier M (2018). 2018 ESC/ESH Guidelines for the management of arterial hypertension. *European Heart Journal*.

[12] Weizhong Z, Haiming S, Ruidong W, Qiwen Y, Zhiguo W, Lin Z (1995). A collaborative study on normal reference values of ambulate blood pressure parameters. *Chinese Journal of Cardiology*.

[13] Yuanming Z (1998). *Handbook of Rating Scales in Psychiatry. 2nd edn*.

[14] Xiangdong W (1999). *Rating Scales for Mental Health*.

[15] Mancia G, Bertinieri G, Grassi G, Parati G, Pomidossi G, Ferrari A (1983). Effects of blood-pressure measurement by the doctor on patient’s blood pressure and heart rate. *The Lancet*.

[16] Kai L, Runyu Y, Xiaoping C (2017). Progress of clinical research on white coat hypertension. *Chinese Journal of Hypertension*.

[17] Pioli MR, Ritter AM, de Faria AP, Modolo R (2018). White coat syndrome and its variations: differences and clinical impact. *Integrated Blood Pressure Control*.

[18] Amigo I, Cuesta V, Fernandez A, Gonzalez A (1993). The effect of verbal instructions on blood pressure measurement. *Journal of Hypertension*.

[19] Smith PA, Graham LN, Mackintosh AF, Stoker JB, Mary DASG (2002). Sympathetic neural mechanisms in white-coat hypertension. *Journal of the American College of Cardiology*.

[20] Caini F, Haiying Z, Hao W (2017). Changes and significances of plasma renin activity, angiotensin II and aldosterone in patients with white-coat hypertension. *Journal of Chinese Practical Diagnosis and Therapy*.

[21] Qi Z, Maojia R, Xiaopeng S, Xingsheng Z (2020). Hypertension with Anxiety and Depression. *Advances in Cardiovascular Diseases*.

[22] Graham N, Smith DJ (2016). Comorbidity of depression and anxiety disorders in patients with hypertension. *Journal of Hypertension*.

[23] Johnson HM (2019). Anxiety and Hypertension: is there a Link? A Literature Review of the Comorbidity Relationship between Anxiety and Hypertension. *Current Hypertension Reports*.

[24] Peiliang L, Yan L (2019). Feasibility of flupentixol melitracine tablets in the adjunctive treatment of essential hypertension with significant white coat effect. *Journal of China Prescription Drug*.

[25] Cobos B, Haskard-Zolnierek K, Howard K (2015). White coat hypertension: improving the patient-health care practitioner relationship. *Psychology Research and Behavior Management*.

[26] Li Y, Buys N, Li Z, Li L, Song Q, Sun J (2021). The efficacy of cognitive behavioral therapy-based interventions on patients with hypertension: a systematic review and meta-analysis. *Preventive Medicine Reports*.

[27] Ziyan W, Guohua Z, Qi H (2021). Research progress of depression and anxiety comorbidity in hypertensive patients. *Chinese Journal of Health Care and Medicine*.

[28] Humbert X, Fedrizzi S, Alexandre J, Menotti A, Manrique A, Laurenzi M (2019). Impact of Sex on Office White Coat Effect Tail: Investigating Two Italian Residential Cohorts. *Scientific Reports*.

[29] Feitosa ADM, Mota-Gomes MA, Barroso WS, Miranda RD, Barbosa ECD, Pedrosa RP (2020). Relationship between office isolated systolic or diastolic hypertension and white-coat hypertension across the age spectrum: a home blood pressure study. *Journal of Hypertension*.

[30] Oyola MG, Handa RJ (2017). Hypothalamic-pituitary-adrenal and hypothalamic-pituitary-gonadal axes: sex differences in regulation of stress responsivity. *Stress*.

[31] Sze Y, Brunton PJ (2020). Sex, stress and steroids. *European Journal of Neuroscience*.

[32] Hänninen MA, Niiranen TJ, Puukka PJ, Kesäniemi YA, Kähönen M, Jula AM (2013). Target organ damage and masked hypertension in the general population: the Finn-Home study. *Journal of Hypertension*.

[33] Ming Y, Hui W, Shuguo L, Juanjuan D, Zhengting Z, Dan Z (2018). The relationship between homocysteine and ambulatory arterial stiffness index in very elderly patients with white coat hypertension. *Bachu Medical Journal*.

[34] Ying Y (2019). Psychological problems and nursing measures of senile patients with chronic diseases. *Chinese Journal of Convalescent Medicine*.

